# Preparation and Flame Retardant Properties of Calcium–Aluminium Hydrotalcite with Root Cutting Silicate Layers as Bamboo Flame Retardants

**DOI:** 10.3390/ma14237319

**Published:** 2021-11-29

**Authors:** Ailian Hu, Chungui Du, Yating Hua, Yingying Shan, Chunlin Liu, Shiqin Chen, Qi Li, Hongwei Yu

**Affiliations:** College of Chemistry and Materials Engineering, Zhejiang A & F University, Hangzhou 311300, China; hal15857832323@163.com (A.H.); artyhuahtl@163.com (Y.H.); syy15968566686@163.com (Y.S.); eustaceweaver7187@gmail.com (C.L.); 18768107239@163.com (S.C.); LQ950011@163.com (Q.L.)

**Keywords:** layered double hydroxides (LDHs), root cutting silicate layer, coprecipitation method, preparation, representation, flame retardant, smoke suppression

## Abstract

Bamboo has been widely used in architecture, decoration and other fields because of its advantages of short growth period, high strength and degradability. However, bamboo, as a combustible material like wood, are easy to burn and cause building fires. However, the existing bamboo water-based flame retardants have some shortcomings, such as strong hygroscopicity and easy loss, which limits the application of bamboo products. In order to improve the flame retardant performance of bamboo, CaAl-SiO_2_ layered double hydroxide (LDH) as bamboo flame retardant was synthesised by coprecipitation method. The influence of preparation technology on CaAl–SiO_3_–LDH structures and properties as well as the flame retardant and smoke suppression characteristics of flame retardant-treated bamboo was discussed. The results revealed that the crystallisation temperature, crystallisation time and crystallisation concentration of CaAl–SiO_3_–LDHs considerably affected its structure and properties. The optimum technological parameters for preparing CaAl–SiO_3_–LDHs by using the coprecipitation method are as follows: crystallisation temperature of 100 °C, crystallisation time of 9 h and Ca^2+^ solution molar concentration of 0.33 mol/L. Compared with nonflame-retardant wood, CaAl–SiO_3_–LDH flame retardant treatment delayed the peak time of the heat release rate by 20 s and the ignition time by 77.78% and increased the carbon residue rate by 9.54%. This study can provide reference for the research of new flame retardant for bamboo products.

## 1. Introduction

China is a major exporter of bamboo products, and more than 60% of popular bamboo flooring, bamboo furniture, planted bamboo and recombinant bamboo exported to Europe and America originates from China. The quality and processing technology of Chinese bamboo products are the global standard [1]. However, bamboo, as wood, is a combustible material, and bamboo products burn easily and cause fires, which can result in heavy casualties and property loss [2], which considerably limits the application of bamboo products in architecture and decoration [3]. Therefore, increasing the fire resistance of bamboo through flame-retardant treatment is critical.

Currently, phosphorus–nitrogen and nitrogen–phosphorus–boron composites are typically used as bamboo flame retardants. Most of these retardants are inexpensive, water-based inorganic flame retardants that exhibit flame retardant effects, limited pollution and low price [4]. However, water-based inorganic flame retardants present disadvantages, such as strong hygroscopicity, easy loss and easy precipitation from the treated material surface (frost resistance) [5]. Although there are many new flame retardants [6], like, Gurjot S. Dhaliwal et al. [7] prepared soy-based PU foams by add nanoclay (cloisite na+). Dan Zhang et al. [8] designed and obtained a new environmentally friendly organic hybrid flame retardant CA(H_2_PO_4_)_2_@HCCP by surface treatment technology to overcome the shortcomings of water-based inorganic flame retardants, layered double hydroxides (LDHs) were developed. LDHs are typical anionic materials with metal hydroxide as the main layer, and anions and some water molecules embedded in the layers to form a layered structure. LDHs provide an easy regulation of physical and chemical properties and rich hydroxyl groups [9,10]. Because the laminated surface of LDHs is rich in hydroxyl groups, it can form hydrogen bonds with many hydroxyl groups in bamboo products and can be easily attached to bamboo [11]. Therefore, the use of LDHs is a novel method for overcoming the shortcomings of water-based inorganic flame retardants.

The coprecipitation method is a simple and commonly used method to prepare LDHs flame retardant materials [12,13,14]. LDHs with a certain morphology can be prepared at low temperatures by using a simple preparation process. The hydrotalcite product exhibits excellent crystallinity and regular layered structure [15,16], which is beneficial to obtain hydrotalcite with high thermal stability. Because calcium and silicon reserves are abundant, they are cheap [17]. Therefore, the addition of silicate into calcium and aluminium to prepare calcium and aluminium hydrotalcite with a root cutting silicate layer is not only inexpensive but also results in a product with high thermal stability. Thus, hydrotalcite with excellent flame retardant property can be prepared. Therefore, calcium–aluminium hydrotalcite in root cutting silicate layers (CaAl–SiO_3_–LDHs) was prepared using the coprecipitation method and it was used as a flame retardant for bamboo to improve the flame retardant performance of bamboo. The effects of preparation technology on the structure and properties of calcium–aluminium hydrotalcite in root cutting silicate layer and the flame retardant and smoke suppression properties of bamboo treated by CaAl–SiO_3_–LDHs [18] were investigated. The results of this study can provide a reference for the research on new flame retardants for bamboo products.

## 2. Materials and Methods

### 2.1. Materials

Calcium nitrate (Ca(NO_3_)_2_·4H_2_O), aluminium nitrate (Al(NO_3_)_3_·9H_2_O), sodium silicate (Na_2_SiO_3_·9H_2_O) and sodium hydroxide (NaOH) were obtained from Sinopharm Chemical Reagent Co., Ltd., Shanghai, China. Deionised water was used in the synthesis and washing process. The water was boiled and cooled to room temperature for reuse.

### 2.2. Preparation of The CaAl–SiO_3_–LDHs

**Figure 1:**
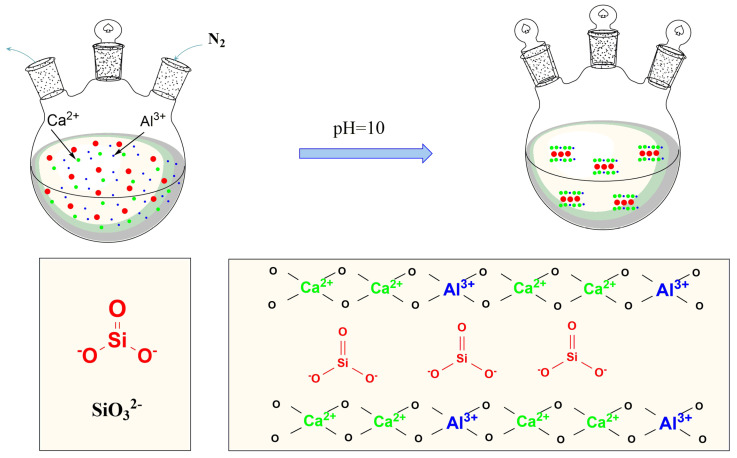
Schematic diagram of synthesis mechanism of CaAl–SiO_3_–LDHs.

The general formula of hydrotalcite is $\left[M_{1-x}^{2+}M_{x}^{3+}{\left(OH\right)}_{2}\right]\left(A_{x/n}^{n-}\right)\cdot mH_{2}O$, where *M* refers to layered cation, A refers to *n*-valent interlayer anion, and $x=nM^{2+}/n\left[M^{2+}+M^{3+}\right]$, 0.16 < *x* < 0.33 [19]. Ca(NO_3_)_2_·4H_2_O and Al(NO_3_)_3_·9H_2_O were weighed and added in the molar ratio of Ca^2+^: Al^3+^ = 2 [20,21,22]. A certain amount of deionised water was added, and the mixture was dissolved in a beaker to prepare a mixed salt solution. A corresponding amount of Na_2_SiO_3_·9H_2_O was weighed and added to deionised water. Ca^2+^ solutions with molar concentrations of 0.13, 0.33, 0.53 and 0.73 mol/L were prepared. A high-concentration NaOH solution was prepared for subsequent use. The sodium silicate solution was added to a 500 mL three-neck flask. The flask was placed in a constant temperature oil bath at 25 °C, and the mixture was stirred. The prepared mixed salt solution and NaOH solution were subjected to a constant pressure dropping funnel. The NaOH solution was added until the pH of the solution in the flask reached 10. Next, the mixed salt solution was added to maintain the pH value of the solution at 10. The prepared white slurry was crystallised at 85, 100 and 120 °C and stirred vigorously at a constant speed for 6, 9 and 12 h and subsequently stored at room temperature. Finally, the cake was filtered, washed to neutrality and dried for 24 h at 80 °C. The synthesis mechanism is illustrated in Figure 1 and the summary table about experimental samples is shown in Table 1.

### 2.3. CaAl–SiO_3_–LDHs Flame-Retardant Treated Bamboo

A CaAl–SiO_3_–LDHs powder was prepared and ultrasonically dispersed in an aqueous solution to obtain 1% suspension, and ultrasonic treatment was performed for 1 h. Then, bamboo chips were placed in the CaAl–SiO_3_–LDHs suspension. Next, the CaAl–SiO_3_–LDHs suspension soaked with bamboo chips is placed in a pressurised tank and sealed. Then samples were vacuumised, and when the vacuum degree reaches 0.085 MPa, kept for 20 min. Next, a pressure of 0.6 MPa was applied. When the pressure reached the preset value, the exhaust valve was opened to relieve the pressure to the preset value. The bamboo pieces were soaked for 3 h. Finally, the bamboo chips were removed, the suspension on the surface was removed with deionised water and bamboo chips were dried in an oven to obtain CaAl–SiO_3_–LDHs flame-retardant bamboo.

### 2.4. Analysis and Testing

#### 2.4.1. X-ray Diffraction (XRD) Analysis

XRD-6000 manufactured by Shimadzu Corporation of Japan was used to analyse the phase composition and crystallisation of the CaAl–SiO_3_–LDHs samples. The X-ray tube was the Cu target, Kα was the radiation source, the tube voltage was 40 Kv, the tube current was 30 mA, the scanning range was 5°–75° (2θ), and the scanning speed was 2 °/min.

#### 2.4.2. Fourier Infrared Spectrum (FT-IR) Analysis

To determine the chemical composition of CaAl–SiO_3_–LDHs prepared under different conditions, the samples dried in the oven at 80 °C were mixed and ground with potassium bromide in a ratio of 1:100. The synthesised CaAl–SiO_3_–LDHs samples were analysed using the Prestige-21 FT-IR spectrometer produced by Shimadzu Corporation of Japan, with a scanning range of 400–4000 cm^−1^.

#### 2.4.3. Cold Field Emission Scanning Electron Microscopy (SEM) Analysis

The morphology and dispersion of CaAl–SiO_3_–LDHs prepared under different conditions were analysed. Cold field emission scanning electron microscope SU8010 produced by Hitachi of Japan was used to analyse CaAl–SiO_3_–LDHs under an accelerating voltage of 25.0 kV.

#### 2.4.4. Thermogravimetry (TG–DTG) Analysis

The thermal stability of CaAl–SiO_3_–LDHs samples was analysed by using the STA 409C thermogravimetric analyser produced by NETZSCH, Germany. The temperature ranged from 35 to 750 °C, the heating rate was 20 °C/min, the sample mass was 6 to 9 mg and during the reaction process, nitrogen is always introduced.

#### 2.4.5. Cone Calorimeter (CONE) Analysis

Nonflame-retardant bamboo chips and flame-retardant treaded bamboo chips were bonded with a small amount of white latex into samples with dimensions of 100 mm × 100 mm × 5 mm (length × width × thickness); the combustion performance of the samples was tested with a cone calorimeter (Fire Testing Technology Ltd., East Grinstead, UK), according to the ISO5660-1 procedure. This is to analyse the influences of CaAl–SiO3–LDHs-flame retardants on the flame-retardant effect of bamboo. The heat radiation power was 50 kW/m^−2^, corresponding to temperature of 728 °C [23,24]. During the experiment, with the exception of the heated surface, all five surfaces of the sample were coated with a tin foil, and subsequently placed into a stainless steel mould box. The sample surface was fixed with barbed wire, and the bottom of the sample was sealed with an asbestos pad. The experimental data were collected, processed and analysed using special cone calorimeter software.

## 3. Results

### 3.1. Effects of Crystallisation Temperature on Structure and Properties of CaAl–SiO_3_–LDHs

**Figure 2:**
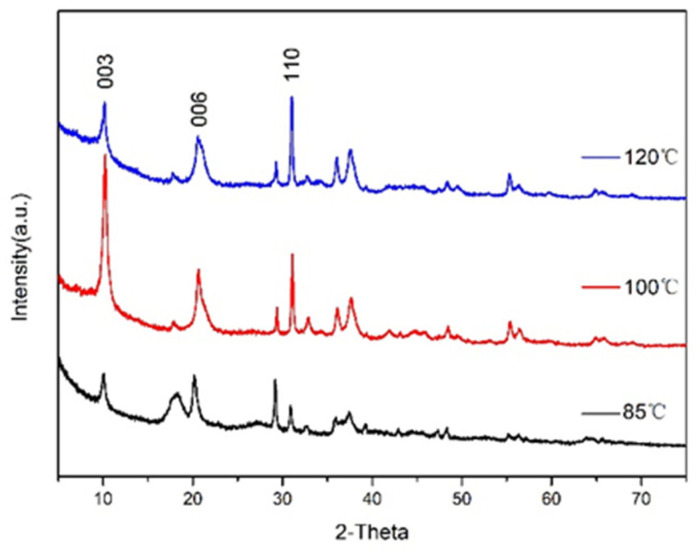
XRD patterns of CaAl–SiO_3_–LDHs prepared at different crystallisation temperatures.

The XRD pattern of CaAl–SiO_3_–LDHs prepared by the crystallisation reaction at 85, 100 and 120 °C for 12 h is displayed in Figure 2.

Several main characteristic peaks of CaAl–SiO_3_–LDHs samples synthesised at 85, 100 and 120 °C corresponded to the characteristic crystal planes (003), (006) and (110) of hydrotalcite. The XRD patterns characteristic peak of CaAl–SiO_3_–LDHs crystallised at 100 °C was the sharpest, and its intensity was the highest, which indicated that the crystal lattice of the sample was the most complete, and the crystallinity was the highest. Therefore, 100 °C is the best crystallisation temperature of CaAl–SiO_3_–LDHs.

**Figure 3:**
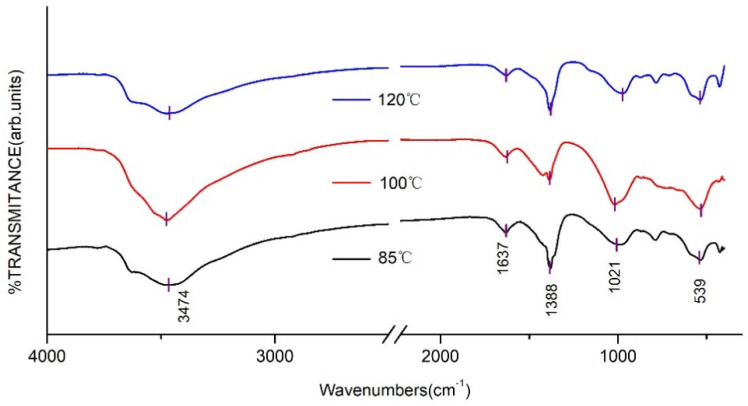
FT-IR spectrum of CaAl–SiO_3_–LDHs prepared at different crystallisation temperatures.

The FT-IR spectra of CaAl–SiO_3_–LDHs prepared at various crystallisation temperatures are displayed in Figure 3.

Figure 3 reveals that the FT-IR absorption peaks of CaAl–SiO_3_–LDHs prepared at various crystallisation temperatures are similar. The absorption peak near 3474 cm^−1^ was related to the stretching vibration of hydroxyl groups of silicate calcium–aluminium hydrotalcite laminate or hydroxyl groups that physically adsorb water molecules. The absorption peak near 1637 cm^−1^ was caused by the tensile vibration of hydroxyl groups in crystallised water. The absorption peaks at 1021 and 539 cm^−1^ were caused by the vibration of silicate ions [25], which indicated that silicate was successfully embedded into hydrotalcite. The appearance of the shoulder peak at 1388 cm^−1^ was attributed to CO_3_^2−^,which indicated that CO_3_^2−^ was doped in hydrotalcite, which could be related to the strong adsorption capacity of calcium–aluminium hydrotalcite for CO_2_ caused by the adsorption of CO_2_ when the sample was exposed to air after preparation [26].

**Figure 4:**
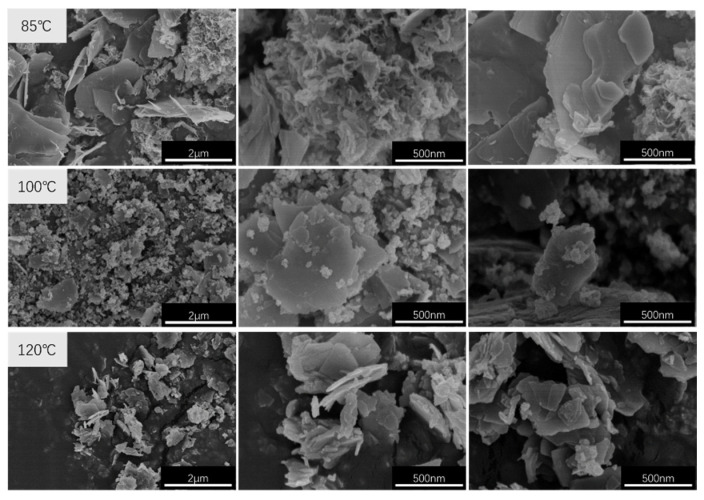
SEM images of CaAl–SiO_3_–LDHs samples prepared at different crystallisation temperatures.

The SEM images of CaAl–SiO_3_–LDHs prepared at crystallisation temperatures of 85, 100 and 120 °C are displayed in Figure 4.

Figure 4 reveals that all the CaAl–SiO_3_–LDHs samples exhibit obvious layered structure, which shows the characteristic structure of hydrotalcite. The samples prepared at crystallisation temperatures of 85 °C and 100 °C were unevenly distributed and exhibited two morphologies, namely larger lamellar and smaller lamellar. But the samples prepared at crystallisation temperatures of 85 °C, the smaller lamellar structure was present in a larger proportion; among the samples prepared at 100 °C, the smaller layered structure accounts for a large proportion. However, the samples prepared at 120 °C exhibited a uniform morphology distribution, but its lamellar size was typically large.

**Figure 5:**
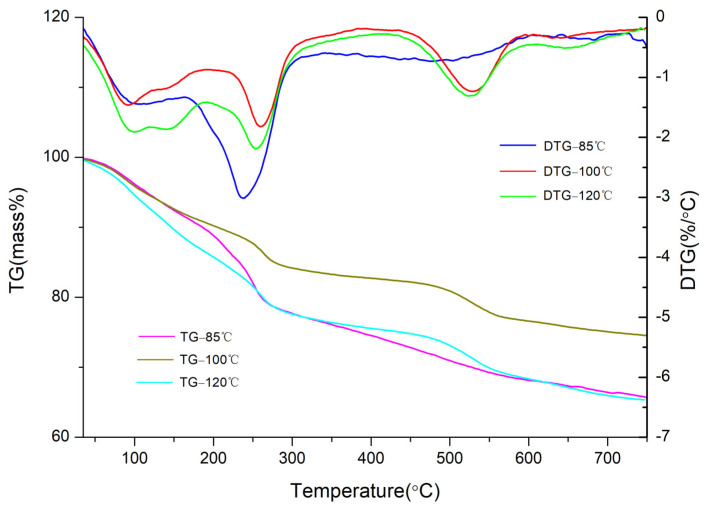
TG-DTG image of CaAl–SiO_3_–LDHs samples prepared at different crystallisation temperatures.

The TG–DTG curves of CaAl–SiO_3_–LDHs samples prepared at various crystallisation temperatures are displayed in Figure 5.

Thermal weightlessness can be clearly categorised into three weightless stages (Figure 5). The first weight loss stage was attributed to the loss of water molecules and physically adsorbed water in the interlayer of the sample. The second weight loss stage was attributed to the detachment of cationic lamellar hydroxyl groups in the sample and the third weight loss stage was attributed to the pyrolysis of interlayer anions in the sample [27]. In the first weight loss stage, the weight loss rate, maximum weight loss rate and temperature to reach the maximum weight loss rate of the three samples were similar, indicating that the amounts of crystal water and adsorbed water of the three samples were similar. In the second weight loss stage, the thermal weight loss of CaAl–SiO_3_–LDHs samples crystallised at 85 °C occurred at 165–299 °C, with a weight loss rate of 13%. The weight loss rate and weight loss temperature of the samples crystallised at 85 °C were higher and lower, respectively, than the other two samples, which indicated that the layered structure was unstable and numerous hydroxyl groups were lost. In the third stage of thermal weight loss, the CaAl–SiO_3_–LDHs samples crystallised at 100 °C exhibited thermal weight loss at 480–564 °C, with a weight loss rate of 5%. Weight loss temperature, the weight loss peak and the thermal stability of the CaAl–SiO_3_–LDHs samples crystallised at 100 °C were higher, narrower and better than those of the other samples. Therefore, the composition and distribution of interlayer anions in CaAl–SiO_3_–LDHs samples crystallised at 100 °C were uniform. Thus, 100 °C was the best crystallisation temperature for preparing CaAl–SiO_3_–LDHs.

### 3.2. Effect of the Crystallisation Time on the Structure and Properties of CaAl–SiO_3_–LDHs

**Figure 6:**
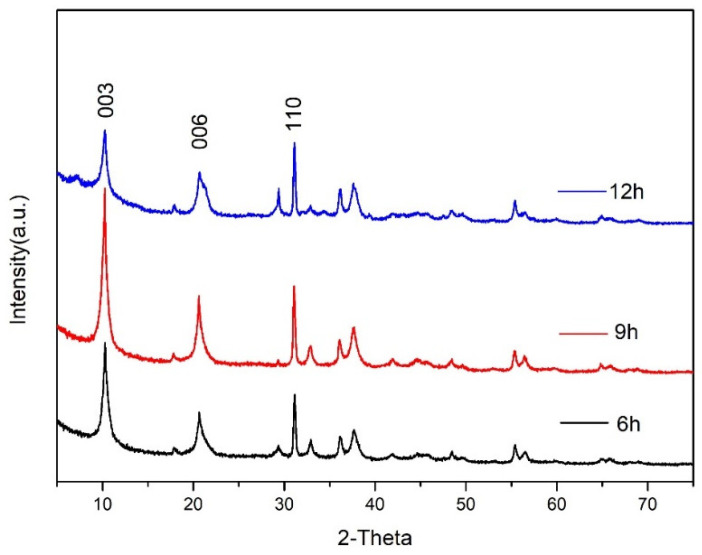
XRD patterns of CaAl–SiO_3_–LDHs prepared at different crystallisation time.

The XRD patterns of CaAl–SiO_3_–LDHs prepared by crystallisation reaction at 100 °C for 6, 9 and 12 h are displayed in Figure 6.

Figure 6 reveals that the main characteristic peaks of the synthesised CaAl–SiO_3_–LDHs sample are similar to the characteristic crystal planes (003), (006) and (110) of hydrotalcite. This phenomenon indicated that the synthesised sample has a typical hydrotalcite lattice structure. The difference in location of each characteristic crystal plane between various samples was not more than 0.2, which indicated that the crystal lattice structure of the synthesised samples was stable when the crystallisation temperature was 100 °C, proving that 100 °C was the best crystallisation temperature for synthesising CaAl–SiO_3_–LDHs. Among all the samples, the XRD characteristic peaks intensity of the samples with crystallisation time of 9 h was the highest and sharpest, which indicated that the samples with the crystallisation time of 9 h exhibited the highest crystallinity.

**Figure 7:**
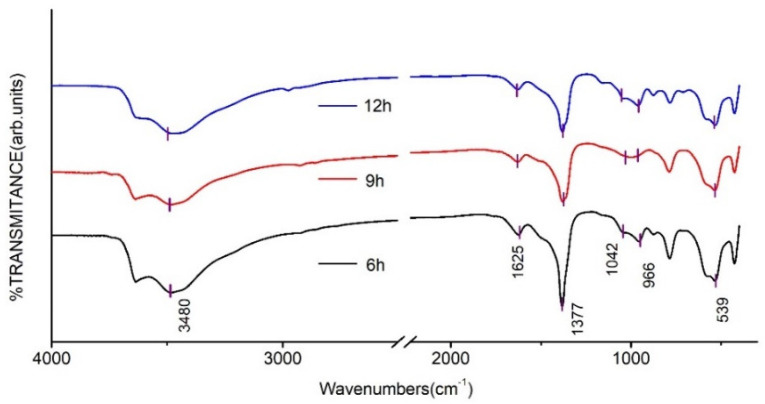
FT-IR spectrum of CaAl–SiO_3_–LDHs prepared at different crystallisation time.

The FT-IR spectra of CaAl–SiO_3_–LDHs prepared by crystallisation reaction at 100 °C for 6, 9 and 12 h are displayed in Figure 7.

The FT-IR absorption peaks of several CaAl–SiO_3_–LDHs samples were similar (Figure 7). The absorption peak near 3480 cm^−1^ was related to the stretching vibration of hydroxyl groups in silicate calcium–aluminium hydrotalcite laminate or hydroxyl groups that physically adsorbed water molecules, whereas the absorption peak near 1625 cm^−1^ was caused by the stretching vibration of hydroxyl groups in crystallised water. The peaks at 1042–966 and 539 cm^−1^ were caused by the vibration of silicate ions, which indicated that silicate was successfully embedded in hydrotalcite. The peak of tensile vibration of CO appeared at 1377 cm^−1^, which indicated that the hydrotalcite was doped with CO_3_^2−^ and could be related to the strong adsorption capacity of calcium–aluminium hydrotalcite for CO_2_ caused by the adsorption of CO_2_ after the sample was prepared and exposed to air.

**Figure 8:**
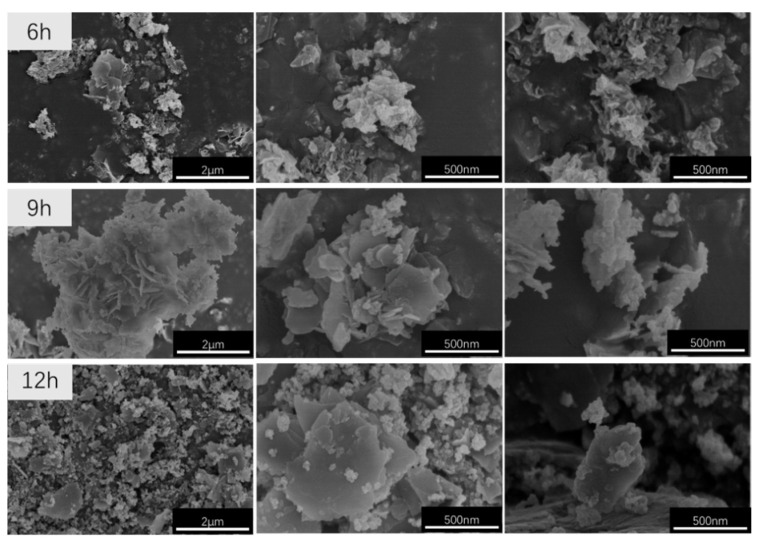
SEM images of CaAl–SiO_3_–LDHs samples prepared at different crystallisation time.

The SEM images of the CaAl–SiO_3_–LDHs samples prepared at 100 °C and various crystallisation times are displayed in Figure 8.

Figure 8 reveals that every CaAl–SiO_3_–LDHs sample exhibits an obvious layered structure. Among them, the samples prepared at crystallisation times of 6 and 12 h were not uniform in morphology and exhibited two or more large-layered structures, namely larger- and smaller-layered structures. Furthermore, the sizes of various layered structures differed considerably, and some even varied by >10 times. However, when the crystallisation time was 9 h, the morphology of CaAl–SiO_3_–LDHs samples was uniform and exhibited almost no difference. Therefore, the crystallisation time of 9 h was the best time for the preparation of CaAl–SiO_3_–LDHs.

**Figure 9:**
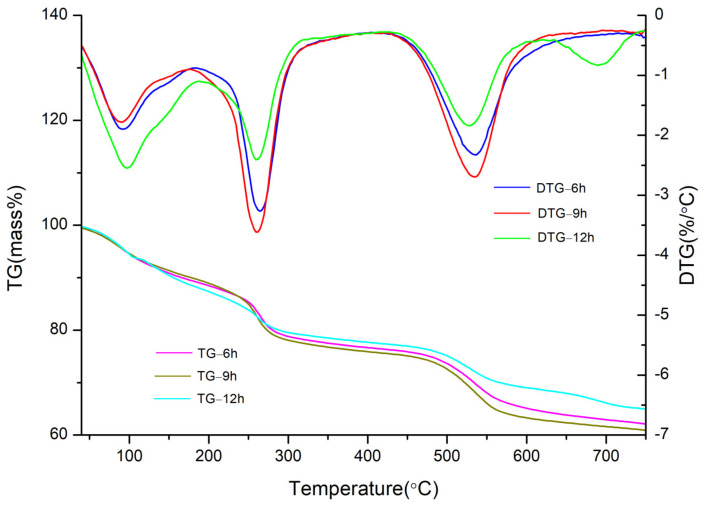
TG-DTG of CaAl–SiO_3_–LDHs samples prepared at different crystallisation time.

The TG–DTG curves of CaAl–SiO_3_–LDHs samples prepared at crystallisation times of 6, 9 and 12 h respectively, are displayed in Figure 9.

Figure 9 reveals that the thermal weight loss of CaAl–SiO_3_–LDHs samples prepared at various crystallisation times can be categorised into three stages, which indicated that the thermal weight loss law of the prepared samples conformed to the three-stage thermal weight loss law of hydrotalcite, in which the sample prepared after crystallisation for 6 h exhibited the smallest weight loss, whereas those prepared after crystallisation for 9 h exhibited the largest weight loss and the weight loss in the second and third stages was higher than that in other stages. The relative contents of –OH and anions in CaAl–SiO_3_–LDH samples were higher in the samples crystallised for 9 h than those in other samples. The weight loss peak of the DTG curve of the sample prepared by crystallisation for 9 h was the narrowest, which indicated that the sample were uniform [28], thus verifying the conclusions of SEM images. Moreover, the sample prepared by crystallisation for 9 h exhibited a slightly higher thermal loss temperature than other sample, which indicated their thermal stability was good. Thus, when CaAl–SiO_3_–LDHs was prepared by the coprecipitation method, the best crystallisation time is 9 h.

### 3.3. Effect of the Crystallisation Concentration on the Structure and Properties of CaAl–SiO_3_–LDHs

**Figure 10:**
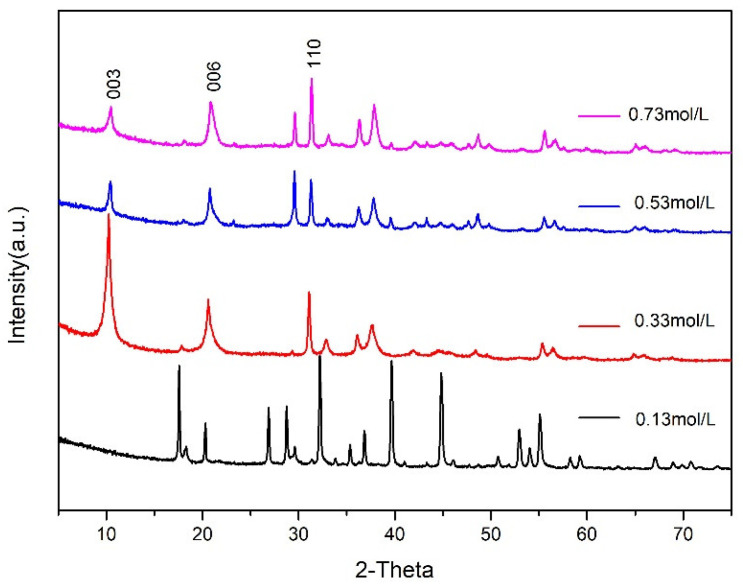
XRD patterns of CaAl–SiO_3_–LDHs prepared at different Ca^2+^ molar concentrations.

The effects of Ca^2+^ molar concentrations of 0.13, 0.33, 0.53 and 0.73 mol/L on CaAl–SiO_3_–LDHs prepared at crystallisation temperature of 100 °C and a crystallisation time of 9 h were investigated. The XRD patterns of the prepared samples are displayed in Figure 10.

Figure 10 reveals that structural characteristic peaks of the hydrotalcite samples with Ca^2+^ molar concentration of 0.13 mol/L are not obvious. However, the main characteristic peaks of the CaAl–SiO_3_–LDHs samples with Ca^2+^ molar concentrations of 0.33, 0.53 and 0.73 mol/L differed from the characteristic crystal planes (003), (006) and (110) of hydrotalcite. The XRD patterns of CaAl–SiO_3_–LDHs synthesised with the molar concentration of Ca^2+^ of 0.33 mol/L exhibited the sharpest characteristic peak and the highest intensity, which indicated that the sample exhibited the most complete lattice and the highest crystallinity. Therefore, the molar concentration of Ca^2+^ of 0.33 mol/L is the optimal molar concentration for synthesising CaAl–SiO_3_–LDHs. Due to the fact that the sample with the concentration of 0.13 does not have the structural characteristics of hydrotalcite, it will not be further explored.

**Figure 11:**
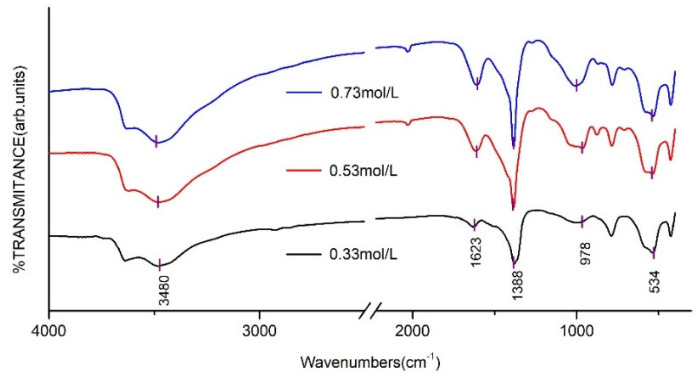
FT-IR spectrum of CaAl–SiO_3_–LDHs prepared at different Ca^2+^ molar concentrations.

The FT-IR spectra of CaAl–SiO_3_–LDHs prepared with the molar concentrations of 0.33, 0.53 and 0.73 mol/L are displayed in Figure 11.

The positions of FT-IR absorption peaks of three CaAl–SiO_3_–LDHs samples are similar. The absorption peak near 3480 cm^−1^ was related to the stretching vibration of hydroxyl groups in silicate calcium–aluminium hydrotalcite laminate or hydroxyl groups that physically adsorb water molecules, whereas the absorption peak near 1623 cm^−1^ was caused by the stretching vibration of hydroxyl groups in crystallised water. The vibration peaks of silicate ions appeared at 978 and 539 cm^−1^, which indicated that silicate ions were successfully embedded in hydrotalcite. The peak of tensile vibration of CO appeared at 1388 cm^−1^, which indicated that CO_3_^2−^ was doped in hydrotalcite.

**Figure 12:**
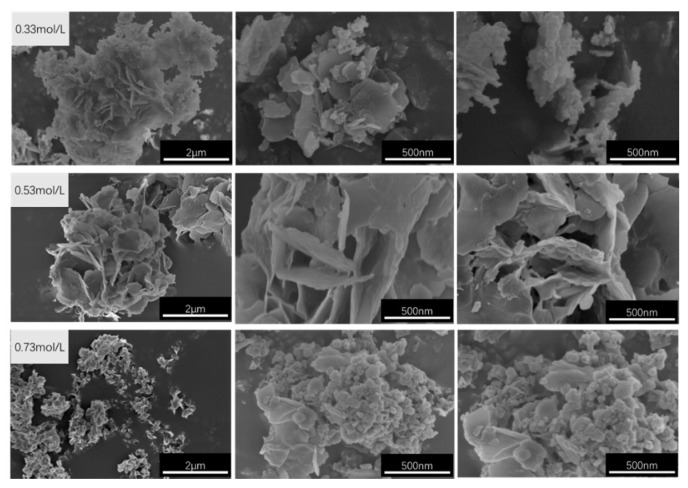
SEM images of CaAl–SiO_3_–LDHs samples prepared at different Ca^2+^ molar concentrations.

The SEM images of CaAl–SiO_3_–LDHs prepared with molar concentrations of 0.33, 0.53 and 0.73 mol/L are displayed in Figure 12.

Figure 12 reveals that the CaAl–SiO_3_–LDHs samples prepared at concentrations of 0.33 and 0.53 mol/L exhibited an obvious layered structure, which is consistent with XRD analysis results. The sample with the concentration of 0.53 mol/L exhibited a uniform appearance, but its size was large. Thus, 0.33 mol/L is the best concentration for preparing CaAl–SiO_3_–LDHs because the sample with 0.33 mol/L was the most uniform in morphology and size.

**Figure 13:**
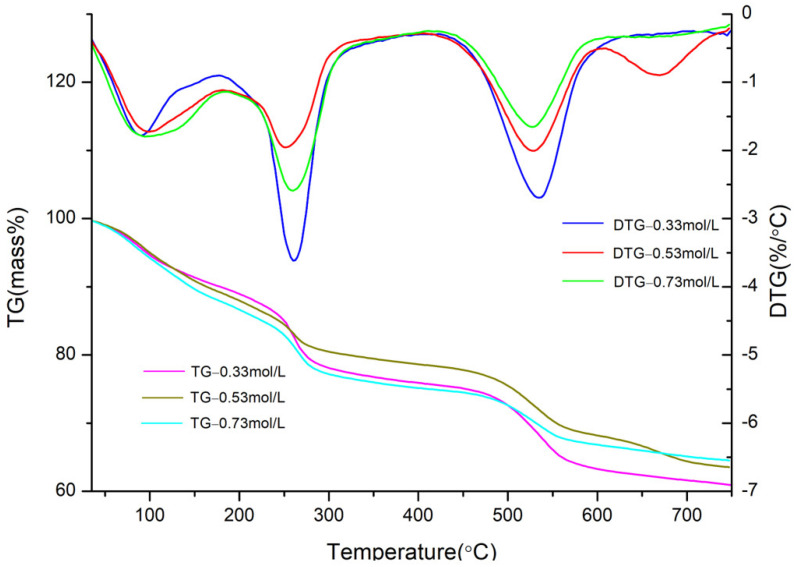
TG-DTG image of CaAl–SiO_3_–LDHs samples prepared at different Ca^2+^ molar concentrations.

The TG–DTG curves of CAl–SiO_3_–LDHs prepared with molar concentrations of 0.33, 0.53 and 0.73 mol/L are displayed in Figure 13.

Figure 13 reveals that the weight loss of samples with molar concentrations of 0.33, 0.53 and 0.73 mol/L can be categorised into three stages, which indicates that the law of weight loss of the prepared samples accords with the law of three-stage weight loss of hydrotalcite. However, the weight loss peak of the DTG curve of the sample prepared with the concentration of 0.33 mol/L was the narrowest, which indicated that the sample was more uniform, and its thermal weight loss temperature was slightly higher than that of other samples, and its thermal stability was satisfactory. The optimum Ca^2+^ concentration for preparing CaAl–SiO_3_–LDHs by coprecipitation was 0.33 mol/L.

### 3.4. Heat Release Rate (HRR)

**Figure 14:**
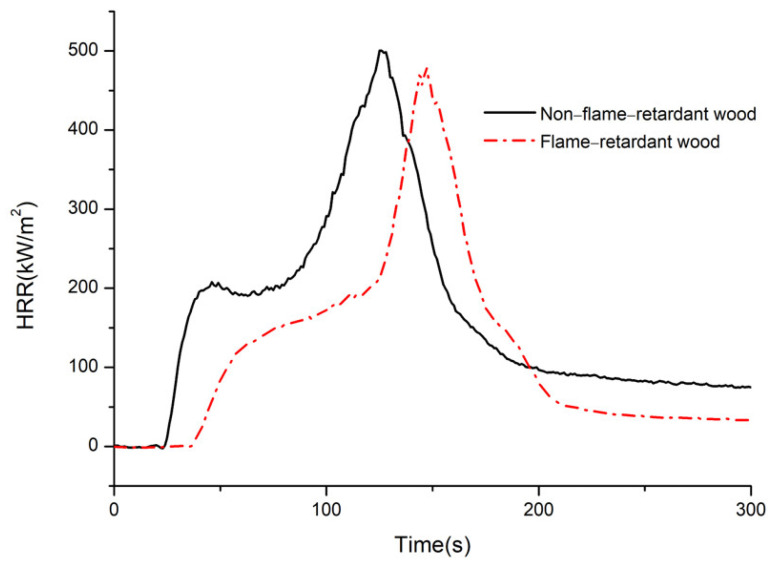
HRR of non-flame retardant bamboo and flame retardant bamboo.

The HRR curves of bamboo before and after flame retardant treatment of CaAl–SiO_3_–LDHs are displayed in Figure 14.

The HRR of materials during combustion can reflect the speed and magnitude of heat released from a fire source [29]. The larger the HRR is, the more heat is fed back to the surface of the material, which accelerates the pyrolysis of the material, increases the generation of volatile combustible materials and finally accelerates flame propagation. Lowering the HRR can effectively reduce the fire risk of the material. Two HRR peaks can be observed in the burning process of bamboo. Figure 14 reveals the first HRR peak appears at 45 s, and the first HRR peak of flame-retardant wood is 25% lower than that of nonflame-retardant wood. In nonflame-retardant bamboo chips, the HRR peaks appeared at 125 s. Compared with non-flame-retardant bamboo, and the values of the second HRR peak of treated bamboo decreased by 13.79%, and its arrival time was delayed by 20 s, which indicated that the decomposition or absorption of CaAl−SiO_3_−LDHs reduced the heat generated by bamboo combustion and inhibited bamboo combustion. When the fire was at its peak and the HRR reached its peak, CaAl–SiO_3_–LDHs delayed the arrival time and improved the flame retardancy of bamboo.

### 3.5. Time to Ignition (TTI)

The TTI refers to the continuous ignition time required for the flame to burn on the material surface. The longer the ignition time is, the less likely ignition occurs under experimental conditions, and the better the flame retardance of the material is [30]. In the cone calorimeter, the ignition time of nonflame-retardant bamboo and bamboo embedded with flame retardant was 18 and 32 s, and the ignition time of flame retardant treated wood was delayed by 77.78%. Therefore, the bamboo embedded with CaAl−SiO_3_−LDHs did not easily catch fire, and the flame retardant property of bamboo was considerably improved.

### 3.6. Mass of Residue(Mass)

**Figure 15:**
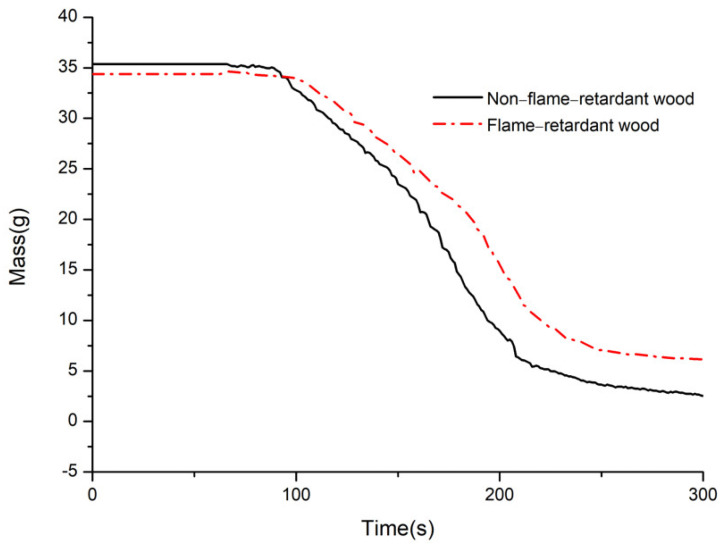
Mass of non-flame retardant bamboo and flame retardant bamboo.

Figure 15 reveals the curve of the residual mass of bamboo before and after flame retardant treatment with CaAl−SiO_3_−LDHs.

The final remaining mass of nonflame-retardant bamboo chips accounted for 8% of the initial mass, whereas that of bamboo chips embedded with flame retardant accounted for 17.54% of the initial mass. Compared with nonflame-retardant bamboo chips, the residue mass of bamboo after treatment improved by 9.54%, which indicates that the addition of CaAl−SiO_3_−LDHs improved the carbon residue rate of bamboo, such that CaAl−SiO_3_−LDHs could promote char formation during combustion.

## 4. Conclusions

(1) The crystallisation temperature of CaAl–SiO_3_–LDHs considerably influenced its structure and properties. The crystallinity and thermal stability of CaAl–SiO_3_–LDHs prepared at 100 °C was the highest.
(2) The crystallisation time of CaAl–SiO_3_–LDHs considerably influenced its structure and properties. The sample prepared at 9 h exhibited high crystallinity, obvious layered structure and the best thermal stability.
(3) The molar concentration of solution of CaAl–SiO_3_–LDHs considerably influenced its structure and properties. When the molar concentration of Ca^2+^ was 0.33 mol/L, the crystallinity of the sample was the highest, the lamellar was the most uniform and the thermal stability is excellent.
(4) The optimum technological parameters for preparing CaAl–SiO_3_–LDHs by the coprecipitation method are as follows: crystallisation temperature 100 °C, crystallisation time 9 h and molar concentration of Ca^2+^ solution 0.33 mol/L. It is not a cheap raw material, but also has high thermal stability.
(5) Compared with the nonflame-retardant bamboo chips, after flame retardant treatment with CaAl–SiO_3_–LDHs, the peak times of HRR and ignition were delayed by 20 s and 77.78%, respectively, and the residual carbon rate was increased by 9.54%, the results show that the addition of CaAl–SiO_3_–LDHs greatly improves the flame retardancy of bamboo.

## Figures and Tables

**Table 1:** Summary of experimental samples.

	Sample Number	Crystallisation Temperatures	Crystallisation Times	Ca^2+^ Molar Concentrations
Samples prepared at different crystallisation temperatures	①	85 °C	12 h	0.33 mol/L
	②	100 °C	12 h	0.33 mol/L
	③	120 °C	12 h	0.33 mol/L
Samples prepared at different crystallisation times	④	100 °C	6 h	0.33 mol/L
	⑤	100 °C	9 h	0.33 mol/L
	⑥	100 °C	12 h	0.33 mol/L
Samples prepared at different Ca^2+^ molar concentrations	⑦	100 °C	9 h	0.13 mol/L
	⑧	100 °C	9 h	0.33 mol/L
	⑨	100 °C	9 h	0.53 mol/L
	⑩	100 °C	9 h	0.73 mol/L

## Data Availability

All data can be found within the manuscript.
